# Complete Genome Sequence of *Enterobacter cloacae* 704SK10, an OXA-48-Encoding Wastewater Isolate

**DOI:** 10.1128/genomeA.00830-17

**Published:** 2017-08-17

**Authors:** Roger Marti, Roger Stephan, Jochen Klumpp, Magdalena Nüesch-Inderbinen, Jörg Hummerjohann, Claudia Bagutti, Katrin Zurfluh

**Affiliations:** a Institute for Food Safety and Hygiene, Vetsuisse Faculty University of Zurich, Zurich, Switzerland; b Institute of Food, Nutrition and Health, ETH Zurich, Zurich, Switzerland; c Division of Food Microbial Systems, Microbiological Safety of Foods of Animal Origin Group, Agroscope, Bern, Switzerland; d Biosafety Laboratory, State Laboratory Basel-City, Basel, Switzerland

## Abstract

Here we present the complete genome sequence of *Enterobacter cloacae* 704SK10, a Swiss wastewater isolate encoding an OXA-48 carbapenemase. Assembly resulted in closed sequences of the 4,876,946-bp chromosome, a 111,184-bp IncF plasmid, and an OXA-48-encoding IncL plasmid (63,458 bp) nearly identical to the previously described plasmid pOXA-48.

## GENOME ANNOUNCEMENT

*Enterobacter cloacae* is ubiquitous in the environment and can be a part of the commensal flora of human and animal gastrointestinal tracts (1). It is also an important opportunistic pathogen associated with nosocomial infections (2) and the third most common species of enterobacteria carrying the class D β-lactamase OXA-48, after *Klebsiella pneumoniae* and *Escherichia coli* (3). OXA-48 is not susceptible to β-lactamase inhibitors, and it hydrolyzes penicillins at high rates and carbapenems at significant, yet low, rates. Although extended-spectrum cephalosporins are not hydrolyzed by this enzyme (4), OXA-48 producers (exclusively enterobacteria [4]) often express extended-spectrum β-lactamases (ESBL) in addition to OXA-48, rendering all β-lactam antibiotics ineffective against such strains (5).

*E. cloacae* 704SK10 was isolated from wastewater near Basel, Switzerland, in December 2015 (6). As the strain tested positive for OXA-48 and was phenotypically resistant to cefotaxime (CTX), it was fully sequenced. We used Pacific Biosciences (PacBio) single-molecule real-time (SMRT) technology RS2 reads with C4/P6 chemistry to sequence the strain (Functional Genomics Center Zurich) and *de novo* assembled the genome using SMRTAnalysis 2.3 (HGAP3 protocol). The MLST-1.8 server (7), ResFinder 2.1 (8), and PlasmidFinder 1.3 (9) (see http://www.genomicepidemiology.org/) were used for initial assessment of sequence type (ST), acquired antibiotic resistances, and plasmid incompatibility (Inc) groups.

The assembly resulted in one chromosome and two plasmids (all sequences are closed), which were automatically annotated using the NCBI Prokaryotic Genome Annotation Pipeline (10). The chromosome is 4,876,946 bp long with a GC content of 55.9% and encodes genes for β-lactam (*bla*_MIR-15_) and fosfomycin (*fosA*) resistance. The strain belongs to ST272, which is not among the high-risk clones identified in a recent study (11). The larger plasmid, p704SK10_1 (IncF), is 111,184 bp in size with a G+C content of 50.9% and encodes no acquired antibiotic resistance genes. The 63,458-bp OXA-48-encoding IncL plasmid p704SK10_2 (G+C content, 51.2%) is nearly identical to pOXA-48 (GenBank accession no. JN626286), the prototype of highly similar IncL plasmids responsible for the dissemination of OXA-48 (5, 12). The two plasmids are 99.7% identical at the nucleotide (nt) level apart from two insertions in p704SK10_2, which both encode insertion element IS1 protein InsB (WP_001119291.1). The first insertion (777 bp) is 163 bp upstream of the gene encoding OXA-48, and the second one (776 bp) is 356 bp downstream of *korC*, disrupting a hypothetical protein.

*E. cloacae* 704SK10 is another isolate that demonstrates the importance and high degree of conservation of pOXA-48-like IncL plasmids in the dissemination of this carbapenemase in enterobacteria.

### Accession number(s).

Sequence and annotation data of the *E. cloacae* 704SK10 genome have been deposited at GenBank under accession numbers CP022148 (chromosome), CP022149 (p704SK10_1), and CP022150 (p704SK10_2). This is the first version of this genome.
